# Improving Cycle Life of Zinc–Air Batteries with Calcium Ion Additive in Electrolyte or Separator

**DOI:** 10.3390/nano13121864

**Published:** 2023-06-15

**Authors:** Donghao Zhang, Wenbin Hu

**Affiliations:** 1 Key Laboratory of Advanced Ceramics and Machining Technology (Ministry of Education), School of Materials Science and Engineering, Tianjin University, No. 135 Yaguan Road, Tianjin 300072, China; donghaozhang@tju.edu.cn; 2 Tianjin Key Laboratory of Composite and Functional Materials, School of Materials Science and Engineering, Tianjin University, No. 135 Yaguan Road, Tianjin 300072, China; 3 Joint School of National University of Singapore and Tianjin University, International Campus of Tianjin University, Fuzhou 350207, China

**Keywords:** zinc–air battery, cycle life, calcium ion additives, electrolyte, separator

## Abstract

The electrolyte carbonation and the resulting air electrode plugging are the primary factors limiting the cycle life of aqueous alkaline zinc–air batteries (ZABs). In this work, calcium ion (Ca^2+^) additives were introduced into the electrolyte and the separator to resolve the above issues. Galvanostatic charge–discharge cycle tests were carried out to verify the effect of Ca^2+^ on electrolyte carbonation. With the modified electrolyte and separator, the cycle life of ZABs was improved by 22.2% and 24.7%, respectively. Ca^2+^ was introduced into the ZAB system to preferentially react with CO_3_^2−^ rather than K^+^ and then precipitated granular CaCO_3_ prior to K_2_CO_3_, which was deposited on the surface of the Zn anode and air cathode to form a flower-like CaCO_3_ layer, thereby prolonging its cycle life.

## 1. Introduction

Zinc–air batteries (ZABs) have been considered for a stationary energy storage application due to their high theoretical energy densities, abundant resources, low cost, and the environmental compatibility of Zn [1,2,3,4,5,6]. Its semi-open structure, however, inevitably led to the carbonation of electrolyte, and carbon dioxide came from the air as well as the oxidization of C element in air cathode at a high discharge voltage according to Equations (1) and (2) [7,8,9]. It would significantly reduce the cycle life of ZABs and restrict further commercialization [7,8,10,11].

(1) $$C\;(s)+4{OH}^{-}(aq)={CO}_{2}\;(g)+2H_{2}O\;(l)+4e^{-}$$

(2) $$2KOH\;(aq)+{CO}_{2}\;(g)=K_{2}{CO}_{3}(s)\;$$

Therefore, mitigating the carbonation of alkaline electrolytes is crucial for the application of alkaline ZABs, and numerous attempts have been carried out to resolve the above issues. An aqueous chloride-based electrolyte has been provided to obtain a long cycle life of over 400 h but with a relatively low overpotential of less than 1.1 V [12]. Various near-neutral electrolytes have also been tested to exhibit better recyclability but suffer from a lower current density and battery capacity due to the inhibition of oxygen evolution reactions (OERs) and the hydrogen evolution reaction (HER) [13,14,15,16,17,18,19,20]. A “water-in-salt” (WIS) gel polymer electrolyte was proposed to achieve a long cycle life, but only with lower operating power [21]. Additionally, both H_2_SO_4_ and ZnSO_4_ were believed to induce the abrupt loss of battery capacity [22,23,24].

The above investigation of existing literature implied that the inefficient inhibition for the carbonation of alkaline electrolytes has severely limited the cycle life of ZABs, and new regulation strategies should be explored. Alkali earth metal cations, such as Ca^2+^ and Mg^2+^, exhibited strong adsorption capacity for carbonate ions compared with K^+^, which means that alkali earth metal cations were more likely to adsorb and bind more carbonate ions than K^+^ in alkaline electrolytes [25,26,27,28,29,30,31,32,33,34,35,36].

Herein, calcium hydroxide (Ca(OH)_2_) additive was introduced to modify the electrolyte as well as the membrane by the addition of Ca^2+^, which were both assembled in the batteries to evaluate its effect. A series of charge–discharge cycle tests were performed, and then each component of the cycled ZAB was also analyzed, including the anode, air cathode, and separator. The morphology and structure were systematically characterized by X-ray diffraction (XRD), Fourier-transform infrared (FTIR), and scanning electron microscopy (SEM). Electrochemical measurements were also conducted to investigate the performance evolution of the air cathode after cycling. In addition, the conductivity of the electrolyte and the concentration of CO_3_^2−^ were also measured to facilitate the analysis of the mechanism of additives.

## 2. Experimental Section

### 2.1. Materials

The Zn anode was an 80 × 40 × 1 mm zinc sheet, ground with 800 and 1500 mesh sandpapers in sequence, cleaned and dried with deionized water. Commercial Co_3_O_4_/CB air cathode with a 4.5 mg cm^−2^ mass-loading of submicron-scaled Co_3_O_4_ (300 nm) powder was selected, where carbon black (CB, XC-72R) powders and polytetrafluoroethylene (PTFE) were used as the conductive additive and the binders, respectively [37]. A basic electrolyte consisting of 6 M KOH and 0.2 M ZnO with 0.2 M Ca(OH)_2_ was used as the additive. In addition, Ca(OH)_2_ was added to the α-Al_2_O_3_ ceramic separator to achieve a modified separator, and the detailed composition is listed in Table 1.

**Figure 1:**
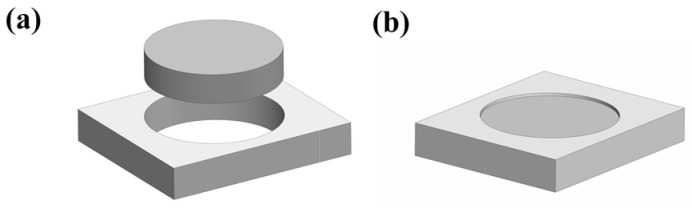
Schematic of the separator preparation mold. (**a**) Schematic diagram of each component, (**b**) Schematic diagram of the assembled mold.

Figure 1 shows the schematic of the separator preparation mold, which consists of two parts, as shown in Figure 1a, and Figure 1b exhibits the schematic after assembly. The slurry was dropped onto polypropylene (pp) film on the mold surface and then dried and turned over to repeat the above process until a satisfactory separator was obtained.

### 2.2. Design and Assembly of ZAB

**Figure 2:**
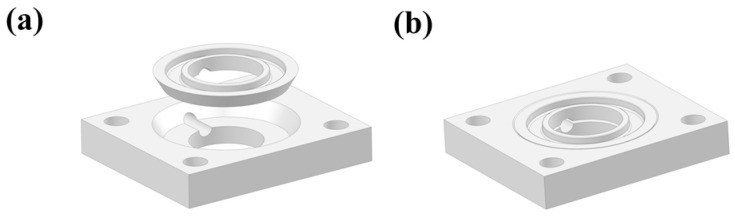
Schematic of electrolytic cell mold that can be installed with a separator. (**a**) Schematic diagram of each component, (**b**) Schematic diagram of the assembled mold.

A typical ZAB mold was applied in this work with the distance between the air cathode and the Zn anode of 1 cm and the active area of the electrode of 4.5 cm^2^ [38,39,40,41,42,43]. In addition, another set of electrolytic cells was installed to explore the effect of the separator on the cycle life of ZABs, as shown in Figure 2, which consisted of an electrolytic cell and a separator fixing plate (Figure 2a). When installing (Figure 2b), the separator was placed into the electrolytic cell and then pressed tightly with the separator fixing plate. When assembling the battery, both sides of the electrolytic cell were fastened to ensure it was completely sealed without liquid leakage during the test.

### 2.3. Methods

Galvanostatic charge–discharge cycle tests were measured at a current density of 10 mA cm^−2^ with a cut-off voltage higher than 5 V or lower than 0 V, using a battery testing system, CT2001A (LanHe Instrument Technology Co., Ltd., Wuhan, China). The cyclic voltammetry (CV), electrochemical impedance spectroscopy (EIS), and linear sweep voltammetry (LSV) were performed at a scan rate of 2 mV s^−1^ with a voltage range of 0 to ±1 V vs. the saturated calomel electrode (SCE) in the solution of 1 M KOH with O_2_ saturated, which were carried out by electrochemical workstation (PARSTAT 4000A, Princeton Applied Research, Oak Ridge, TN, USA). All the tests were carried out at room temperature (25 °C) in the atmosphere environment.

X-ray diffraction (XRD, Bruker D8 advanced, Bremen, Germany) was used to analyze the phase composition of the electrodes after cycle testing with a scanning angle from 10° to 90°. Surface morphologies of the electrodes were characterized by field-emission scanning electron microscopy (SEM, JSM-7800F, with EDS, JEOL, Tokyo, Japan). Additionally, the composition was analyzed using a Fourier-transform infrared (FTIR) (FTIR-650, Tianjin Guangdong Co., Ltd., Tianjin, China) with a wavelength range from 4000 to 400 cm^−1^ and a resolution of 4 cm^−1^.

The concentration of carbonate ion (CO_3_^2−^) in the electrolyte was measured by chemical titration. The conductivity of the electrolyte was tested by a REX DDSJ-308F conductivity meter with a platinum electrode of 5 × 5 mm at 28.6 °C (obtained from the conductivity meter) in the atmospheric environment of 101.1 kPa.

## 3. Results and Discussion

### 3.1. Charge–Discharge Cycle Performance

**Figure 3:**
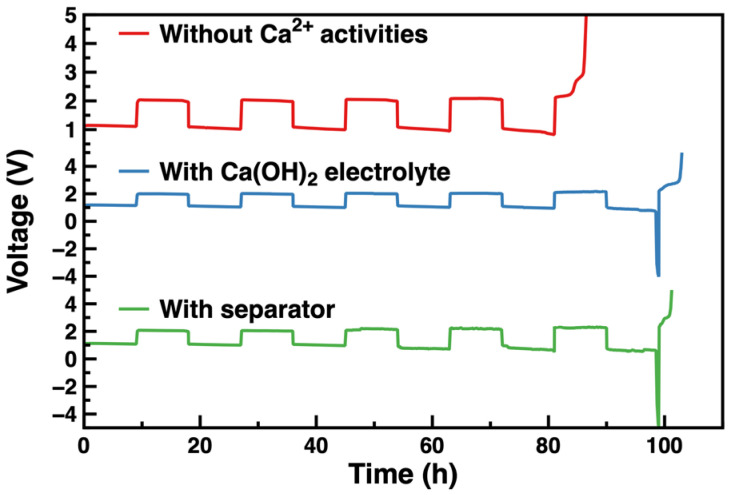
Galvanostatic charge–discharge cycles of zinc–air batteries.

To investigate the effect of Ca^2+^ on the cycle life, ZABs with a modified electrolyte and separator were evaluated with galvanostatic charge–discharge cycle tests; the corresponding results are shown in Figure 3. In addition, a reference ZAB without additives was subjected to the same test as the experimental group. ZABs with a modified electrolyte and separator cycled for 99 h and 101 h, respectively, while the reference one only ran for 81 h, indicating that the addition of the Ca(OH)_2_ additive into the electrolyte and separator improved the cycle life by 22.2% and 24.7%, respectively [11,37,44,45]. It should be noted that the improvement effect of the modified electrolyte is similar to that of the modified separator, owing to the solubility limitation of Ca(OH)_2_ in the aqueous electrolyte.

### 3.2. Morphology and Structure of ZAB Components

To better understand the improvement mechanism of the Ca^2+^ additive on the cycle life of ZABs, the batteries were disassembled to characterize their components, including the Zn anode, air cathodes, electrolyte, and separator.

**Figure 4:**
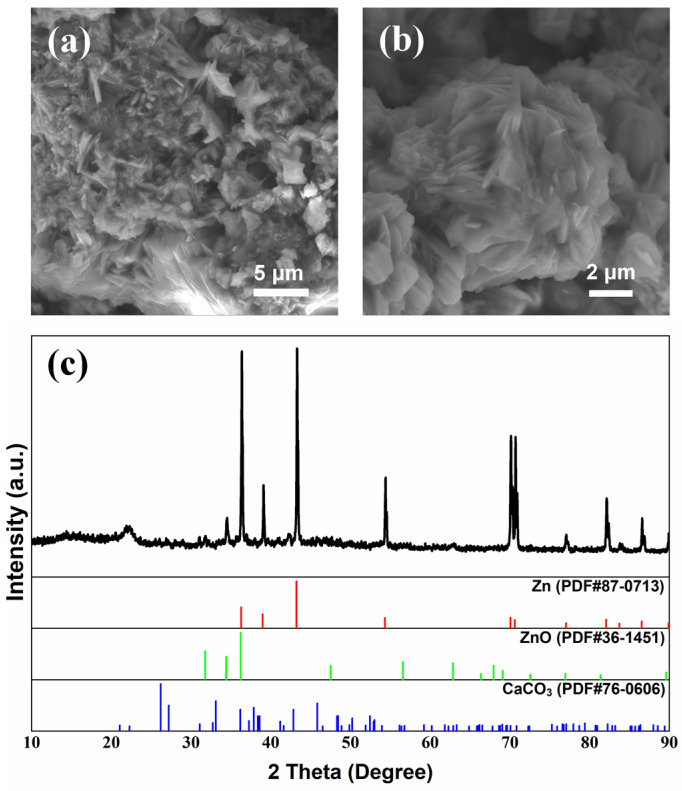
Surface morphology (**a**,**b**) and XRD patterns (**c**) of Zn anode after galvanostatic charge–discharge cycles of ZAB with modified electrolyte.

**Figure 5:**
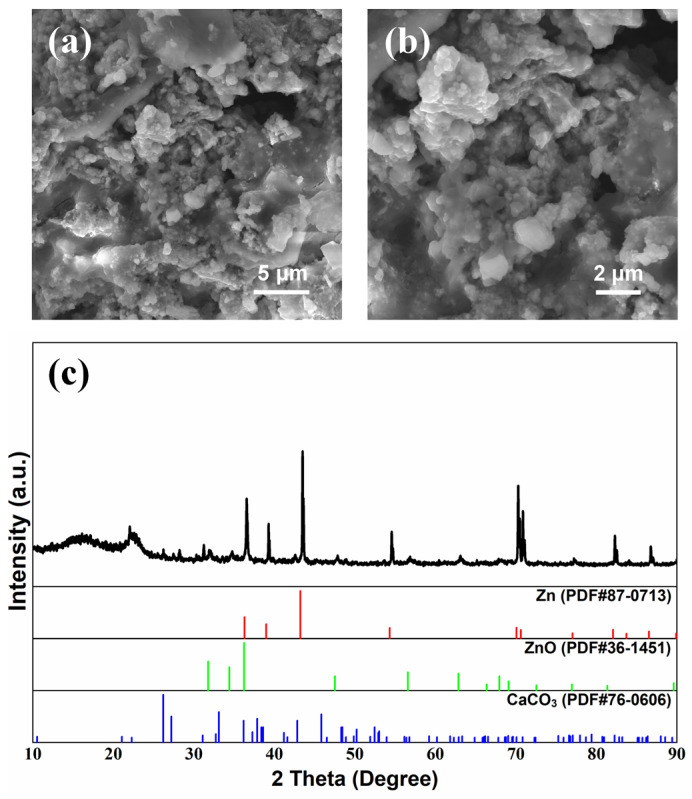
Surface morphology (**a**,**b**) and XRD patterns (**c**) of Zn anode after galvanostatic charge–discharge cycles of ZAB with modified separator.

Surface morphologies of cycled Zn anodes with a modified electrolyte and separator are shown in Figure 4 and Figure 5, respectively, with the corresponding XRD patterns. The surface of the Zn anodes after cycling was relatively flat with a uniformly distributed convex structure (Figure 4a and Figure 5a). After partial amplification, it can be seen in Figure 4b that the convex structure was a flower-like structure, while the 3D microporous structure was formed by alternating stacking of nanoparticle and lamellar structures, as shown in Figure 5b. The corresponding element distributions of cycled Zn anodes are shown in Figures S1 and S2, where the element of Ca was uniformly distributed while elements of Zn and K elements were locally enriched at the surface bumps. The detailed element content results are listed in Tables S1 and S2, and it could be found that the concentration of Ca element on the surface of the Zn anode was relatively low, consistent with the low content of Ca^2+^ additive in the modified electrolyte and separator. In Figure 4c and Figure 5c, the peaks are found to correspond quite well with Zn at 36.3° (0 0 2), 39.0° (1 0 0), 43.2° (1 0 1), and 54.3° (1 0 2) (JCPDS No. 87-0713). Moreover, the peaks matched well at 31.8° (1 0 0) and 36.3° (1 0 1) assigning to ZnO (JCPDS No. 36-1451), indicating the generation of ZnO on the anode surface after cycling [46], confirming the corrosion and passivation of Zn anode during galvanostatic charge–discharge process [47]. Important to note that characteristic peaks of CaCO_3_ (JCPDS No. 76-0606) are detected at 23.0° (0 2 0), 26.2° (1 1 1), 27.2° (0 2 1), 31.1° (0 0 2), 32.7° (1 2 1), 76.6° (2 0 4), 82.3° (1 6 2) and 86.4° (2 3 4), indicating the formation of CaCO_3_ after cycling [26,27,28]. It could be concluded that CO_3_^2–^ was adsorbed and combined with Ca^2+^ to form CaCO_3_ during cycling when adding Ca(OH)_2_ into the electrolyte and separator [26,27,28,29,30], which generated flower-like CaCO_3_ layer to provide the transport channel of OH^−^, thereby improving the cycle life of ZABs [48].

**Figure 6:**
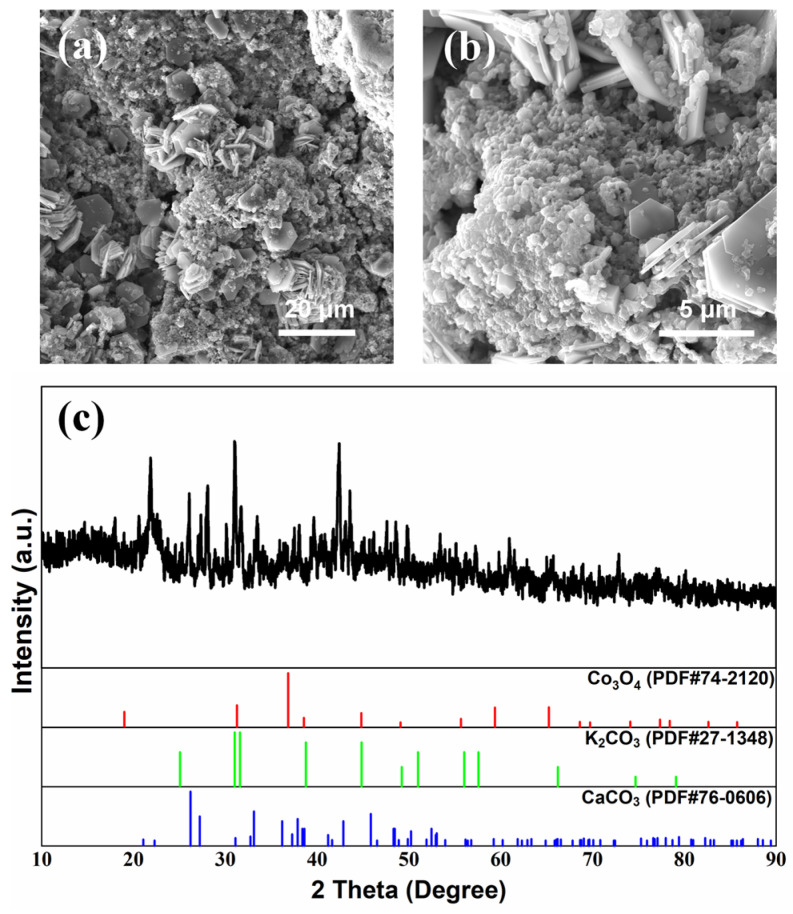
Surface morphology (**a**,**b**) and XRD patterns (**c**) of air cathode after galvanostatic charge–discharge cycles of ZAB with modified electrolyte.

**Figure 7:**
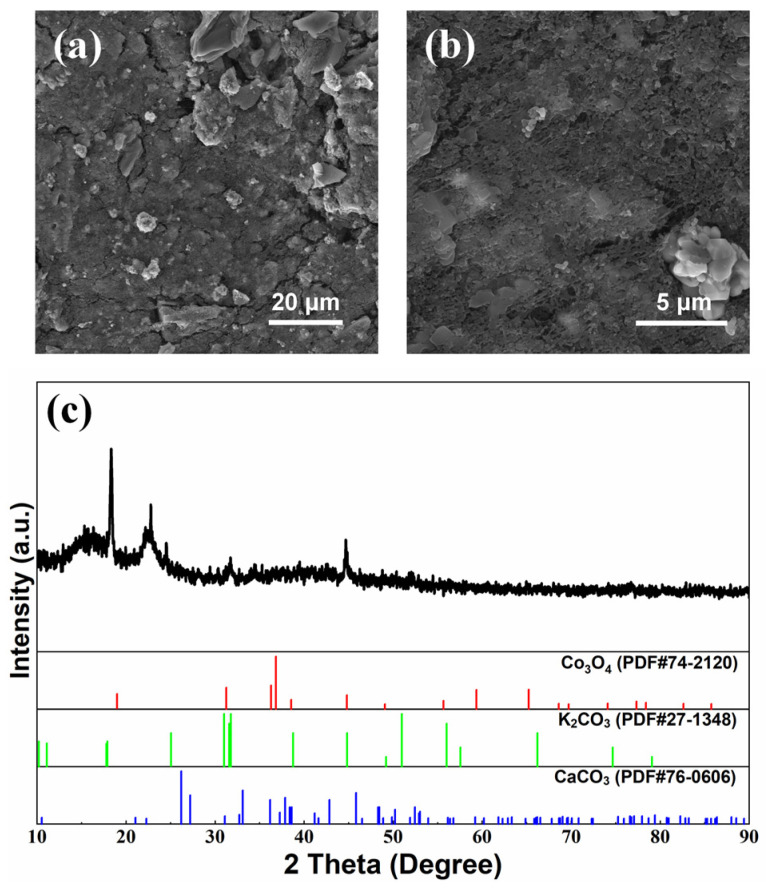
Surface morphology (**a**,**b**) and XRD patterns (**c**) of air cathode after galvanostatic charge–discharge cycles of ZAB with modified separator.

**Figure 8:**
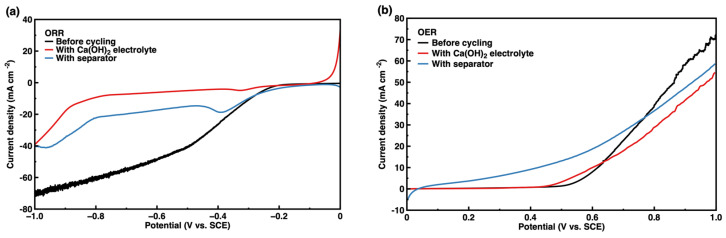
(**a**) ORR and (**b**) OER curves of air cathode after charge-discharge cycles of ZAB.

Surface morphologies of the air cathode after cycling with a modified electrolyte and separator are shown in Figure 6 and Figure 7, respectively, with the corresponding XRD patterns. The lamellar convex structure was evenly distributed on the surface of the air cathode, as shown in Figure 6a and Figure 7a, and the lamellar convex structure and fine particles were alternately distributed, as shown in Figure 6b and Figure 7b. According to the element distribution characterization (Figures S3 and S4) and the detailed element content (Tables S3 and S4), the element of K was densely distributed on the surface of the air cathode with relatively higher coverage, while the element of Ca was enriched around spherical particles in a small amount. In addition, elements of C and O were accumulated at the enrichment sites of K and Ca, and the element of Co was only observed in local areas. In Figure 6c and Figure 7c, the peaks are consistent with Co_3_O_4_ (JCPDS No. 74-2120) at 19.0° (1 1 1), 31.3° (2 2 0), 36.8° (3 1 1), 44.8° (4 0 0), 59.3° (5 1 1), and 65.2° (4 4 0). Additionally, the peaks detected at 25.1° (0 0 2), 31.0° (1 0 2), 31.6° (1 1 0), and 38.8° (2 0 1) are assigned to the K_2_CO_3_ (JCPDS No. 27-1348), and the peaks of CaCO_3_ can be clearly observed at 23.0° (0 2 0), 26.2° (1 1 1), 27.2° (0 2 1), 31.1° (0 0 2), 32.7° (1 2 1), 72.4° (1 2 4), and 77.1° (0 5 3) (JCPDS No. 76-0606). It could be demonstrated that K_2_CO_3_ and CaCO_3_ were generated during charge–discharge cycling [7,8,11,26,27,28,37]. Considering the voltage loss (i.e., *iR* drop) caused by the electrolyte solution between the working electrode and the reference electrode, corrected ORR and OER performance of the cycled air cathode by *iR* compensation was investigated in Figure 8, and both of them were significantly decreased after cycling, and its performance with a modified electrolyte deteriorated more than the one with a modified separator [8,11,26,27,28,29,30,49,50]. After cycling, the micropores of the air cathode could easily be blocked by the generated lamellar K_2_CO_3_, hindering the diffusion of O_2_ through the air cathode to participate in ORR and OER reaction, resulting in the remarkable decrease in ORR and OER performance of air cathode. In comparison, the flower-like porous structure formed by granular CaCO_3_ on the surface of the air cathode could provide gas diffusion channels, and thus both ORR and OER activity of the air cathode could be improved to a certain extent, exhibiting a relatively low decrease in the performance.

The conductivity and concentration of CO_3_^2−^ in the electrolyte were measured to clarify the effect of Ca^2+^, and the detailed values are summarized in Table 2 and Table 3. Prior cycling, the conductivity increased by 2.5% from 63.26 × 10^−2^ S cm^−1^ to 64.85 × 10^−2^ S cm^−1^ when adding 0.2 mol L^−1^ Ca(OH)_2_. After cycling, the conductivity of the modified electrolyte decreased by 36.1% to 41.46 × 10^−2^ S cm^−1^, while the responding value with the modified separator decreased by 39.3% to 38.43 × 10^−2^ S cm^−1^, which was attributed to the increased resistance caused by the consumption of conductive ions as well as the production of CaCO_3_ [26,27,28]. Remarkable changes were detected in the concentration of CO_3_^2−^ when adding Ca(OH)_2_, which significantly decreased from 6.182 mol L^−1^ to 0.800 mol L^−1^ or 0.547 mol L^−1^ after cycling with the modified electrolyte or separator, respectively, as shown in Table 3 [8,11,44]. Ca^2+^ was introduced into the ZAB system to preferentially react with CO_3_^2−^ rather than K^+^, resulting in the precipitation of granular CaCO_3_ prior to K_2_CO_3_ [51], alleviating the blocking effect of micropores on the air electrode surface induced by the carbonation of alkaline electrolyte. Moreover, the modified separator would continuously release Ca^2+^ to the electrolyte to consume CO_3_^2−^ during the charge–discharge process, maintaining a relatively low concentration of CO_3_^2−^ in the electrolyte to achieve sustained long-term improvement [48].

**Figure 9:**
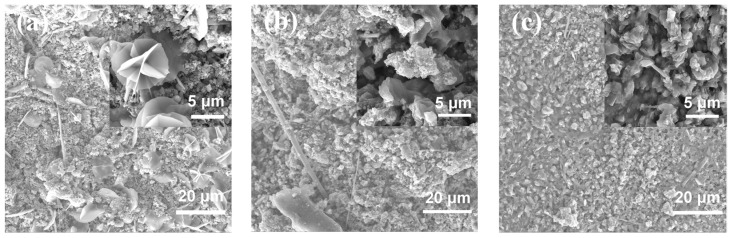
Surface morphology of the separator (**a**) before cycling and after cycling on different sides contacting the (**b**) Zn anode and (**c**) air cathode.

**Figure 10:**
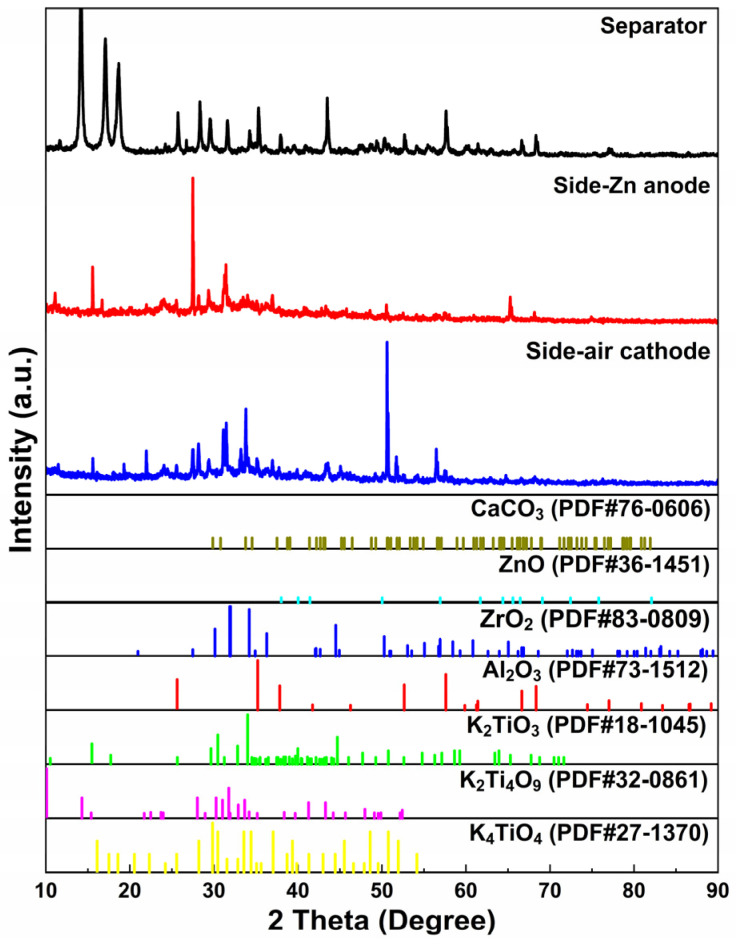
XRD patterns of the fresh separator (black line) before galvanostatic charge–discharge cycles of ZAB, and side to Zn anode (red line) and side to air cathode (blue line) after testing.

Surface morphologies of the modified separator on both sides were investigated before and after cycling, as shown in Figure 9, and the corresponding XRD patterns are exhibited in Figure 10. Prior to cycling, the fresh separator (Figure 9a) is relatively flat, with micropores, whiskers, and nano-spherical structures scattered and uniformly distributed on its surface. After cycling, small spherical structures were observed on the separator when contacting the Zn anode (Figure 9b), forming a loose 3D structure with micropores and whiskers alternately appearing and uniformly distributed on the surface, while at the side contacting the air cathode (Figure 9c), a relatively smoother surface could be found with layered and nano-spherical structures. Combining with the element distribution characterization (Figures S5–S7) and the detailed element content (Tables S5–S7), elements of C, O, Al, K, Ca, and Zn were uniformly distributed on both sides of the separator. Additionally, elements of Ti and Zr were enriched in the surface bumps, and the distribution of Ca, C, and O elements overlapped, indicating the formation of CaCO_3_. According to XRD results in Figure 10, the peaks detected at 31.8° (1 0 0) and 36.3° (1 0 1) at the side of the separator contacting the Zn anode referred to ZnO (JCPDS No.36-1451), and the peaks at 26.2° (1 1 1), 27.2° (0 2 1), 33.1° (0 1 2), 37.8° (1 1 2), 42.9° (1 2 2), and 45.9° (2 2 1) are related with CaCO_3_ (JCPDS No. 76-0606), indicating the generation of ZnO and CaCO_3_ during cycling [26,27,28]. By comparing the infrared spectrum of Ca(OH)_2_ and CaCO_3_ in Figure S8, it could be found that the peak of OH^−^ at 3642 cm^−1^ became weaker with enhanced peaks of C–O at 1432 cm^−1^ and 876 cm^−1^ after cycling, implying the formation of CaCO_3_ to consume CO_2_ by the absorption of Ca^2+^ [29,31,33,34,36,48].

### 3.3. Mechanism of Ca^2+^ Additive to Improve the Cycle Life of ZABs

**Figure 11:**
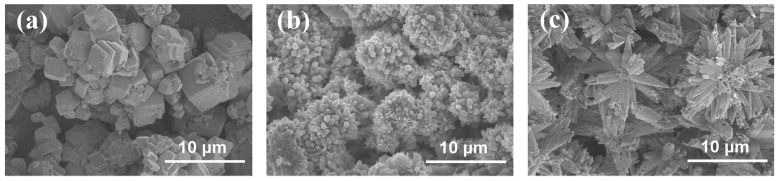
Morphologies of (**a**) CaCO_3_ calcspar and the CaCO_3_ on the surface of (**b**) Zn anode and (**c**) air cathode after galvanostatic charge–discharge cycles of ZAB with modified electrolyte.

To clarify the mechanism of the Ca^2+^ additive in improving the cycle life of ZABs, the morphologies of the CaCO_3_ crystal on the surface of the Zn anode and the air cathode were further characterized by the flower-like structures [48] shown in Figure 11b,c, respectively, exhibiting significant differences from that of the CaCO_3_ calcite particles (Figure 11a). It could be concluded that such flower-like-shaped CaCO_3_ films with large gaps between the crystals provided ion transport channels for electrochemical reactions during the charge and discharge processes, which prevented the blockage of the air cathode by lamellar K_2_CO_3_ crystals [26,27,28,29,30]. This phenomenon occurred mainly due to the strong electrostatic attraction coordinating unsaturated Ca^2+^ in the surface of CaCO_3_ to CO_3_^2−^ [25]. Besides that, CaCO_3_ exhibited relatively lower solubility compared with that of K_2_CO_3_, resulting in the preferential combination of Ca^2+^ with CO_3_^2−^ in the electrolyte to produce CaCO_3_ prior to K_2_CO_3_ [52]. It could alleviate the carbonation of the electrolyte as well as the blockage of the air cathode [26,27,28,29,30], effectively improving the cycle life of ZABs [48].

## 4. Conclusions

In order to alleviate the carbonation of electrolytes to improve the cycle life of ZABs, Ca^2+^ was introduced to modify the electrolyte and the separator. Galvanostatic charge–discharge cycling tests were conducted with morphology and structure analysis of various battery components; subsequently, it could be found that the cycle life of ZABs was improved obviously by 22.2% and 24.7% with the Ca(OH)_2_-modified electrolyte and separator, respectively. When adding Ca(OH)_2_ into the electrolyte and the separator, CO_3_^2−^ was adsorbed to combine with Ca^2+^ to form CaCO_3_ during cycling. The generated CaCO_3_ deposited on the Zn anode and air cathode to produce a flower-like CaCO_3_ layer, which provided ion transport channels for electrochemical reactions during charge–discharge cycles and alleviated the blockage of the air cathode by lamellar K_2_CO_3_ crystals, thereby improving the cycle life of ZABs.

## Figures and Tables

**Table 1:** Composition of the modified separator.

Composition	Weight/g	Purity	Brand
α-Al_2_O_3_	30.0	99.99%	Xinjiang Joinworld
Ca(OH)_2_	10.0	95%	Meryer
ZrO_2_	10.0	99%	Sigma-Aldrich
K_2_O·nTiO_2_	5.0	98%	9dingchem
PVA-124	1.0	AR	Meryer
CMC	1.0	98%	Chemreagent
C_12_H_25_SO_4_Na	0.5	99%	Sigma-Aldrich

**Table 2:** Conductivity of the ZAB electrolyte.

State		Conductivity/×10^−2^ S cm^−1^
Before cycling	Basic electrolyte	63.26
	Modified electrolyte	64.85
After cycling		41.46
	Modified separator	38.43

**Table 3:** Concentration of CO_3_^2−^ in ZAB electrolyte after cycling.

	Without Ca^2+^ Additive	Modified Electrolyte	Modified Separator
Concentration of CO_3_^2−^/mol L^−1^	6.182	0.800	0.547

## Data Availability

Not applicable.

## Supplementary Materials

The following supporting information can be downloaded at: https://www.mdpi.com/article/10.3390/nano13121864/s1. Figure S1: Element distribution of Zn anode after cycle testing of ZAB with modified electrolyte: (a) Ca, (b) Zn, (c) C, (d) O and (e) K. Figure S2: Element distribution of Zn anode after cycle testing of ZAB with modified separator: (a) Ca, (b) Zn, (c) C, (d) O and (e) K. Figure S3: Element distribution of air cathode after cycle testing of ZAB with modified electrolyte: (a) Ca, (b) Co, (c) C, (d) O and (e) K. Figure S4: Element distribution of air cathode after cycle testing of ZAB with modified separator: (a) Ca, (b) Co, (c) C, (d) O and (e) K. Figure S5: Element distribution of the modified separator before cycle testing of ZAB: (a) C, (b) O, (c) Al, (d) K, (e) Ca, (f) Ti and (g) Zr. Figure S6: Element distribution of the modified separator at the side contacting with Zn anode after cycle testing of ZAB: (a) C, (b) O, (c) Al, (d) K, (e) Ca, (f) Ti, (g) Zn and (h) Zr. Figure S7: Element distribution of the modified separator at the side contacting with air cathode after cycle testing of ZAB: (a) C, (b) O, (c) Al, (d) K, (e) Ca, (f) Ti, (g) Co and (h) Zr. Figure S8: FT-IR patterns of the modified separator (a) before and (b) after cycle testing of ZAB. Tabel S1: Element content of Zn anode after cycle testing of ZAB with modified electrolyte. Tabel S2: Element content of Zn anode after cycle testing of ZAB with modified separator. Tabel S3: Element content of air cathode after cycle testing of ZAB with modified electrolyte. Tabel S4: Element content of air cathode after cycle testing of ZAB with modified separator. Tabel S5: Element content of the modified separator before cycle testing of ZAB. Tabel S6: Element content of the modified separator at the side contacting with Zn anode after cycle testing of ZAB. Tabel S7: Element content of the modified separator at the side contacting with air cathode after cycle testing of ZAB.
