# Supplementary material for: Improving Cycle Life of Zinc–Air Batteries with Calcium Ion Additive in Electrolyte or Separator
Source: Nanomaterials (Basel). 2023 Jun 15;13(12):1864. doi: 10.3390/nano13121864 (PMC10302342; doi:10.3390/nano13121864)
Supplement: Supplementary file 1 [file nanomaterials-13-01864-s001.zip › nanomaterials-2417072-supplementary.pdf]

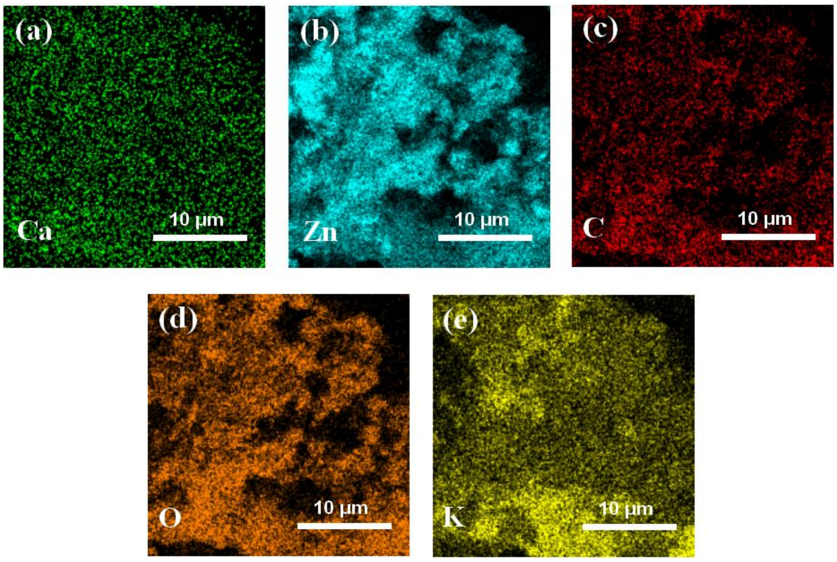

Figure S1. Element distribution of Zn anode after cycle testing of ZAB with modified electrolyte: (a) Ca, (b) Zn, (c) C, (d) O and (e) K.

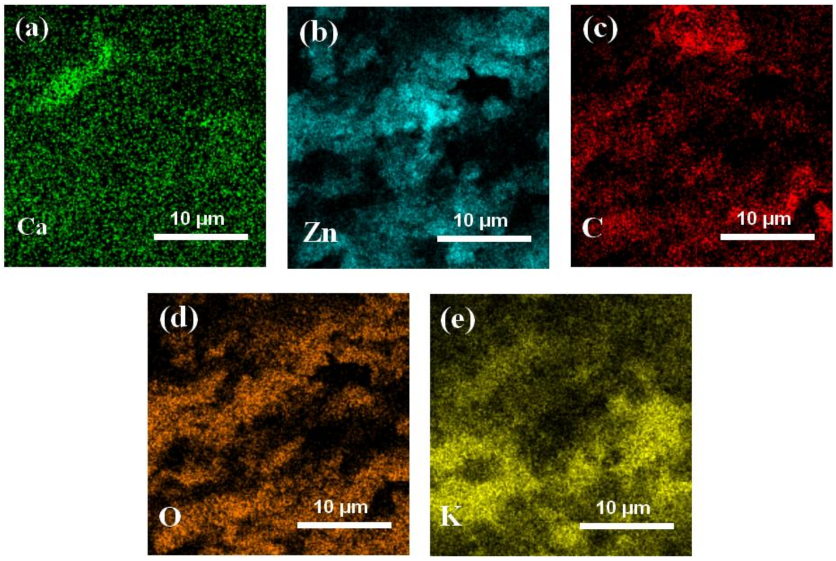

Figure S2. Element distribution of Zn anode after cycle testing of ZAB with modified separator: (a) Ca, (b) Zn, (c) C, (d) O and (e) K.

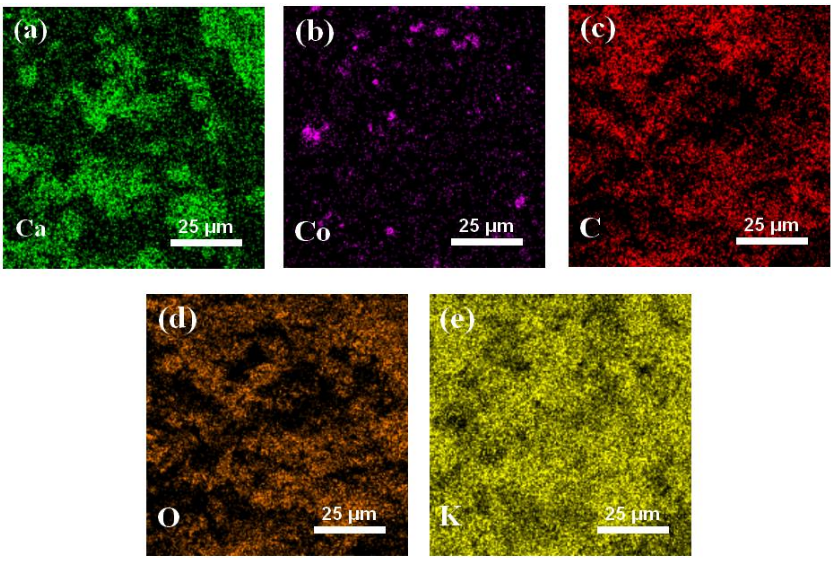

Figure S3. Element distribution of air cathode after cycle testing of ZAB with modified electrolyte: (a) Ca, (b) Co, (c) C, (d) O and (e) K.

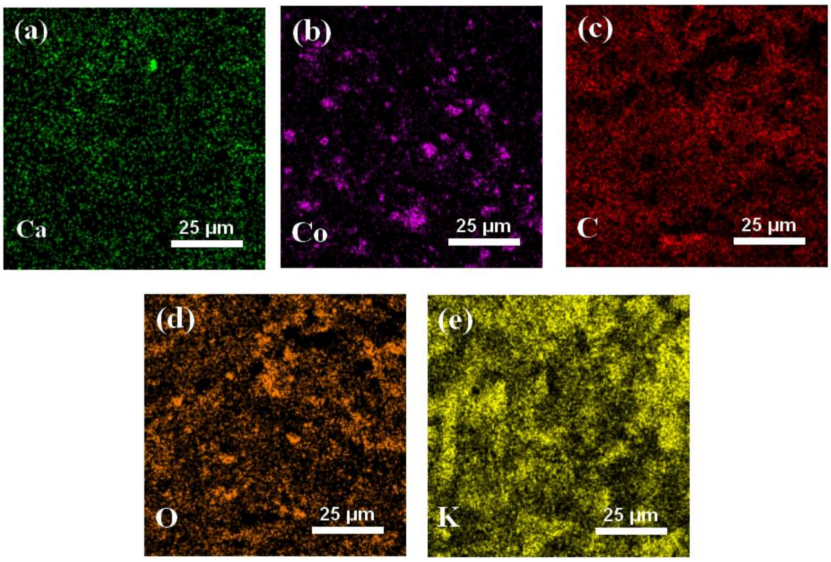

Figure S4. Element distribution of air cathode after cycle testing of ZAB with modified separator: (a) Ca, (b) Co, (c) C, (d) O and (e) K.

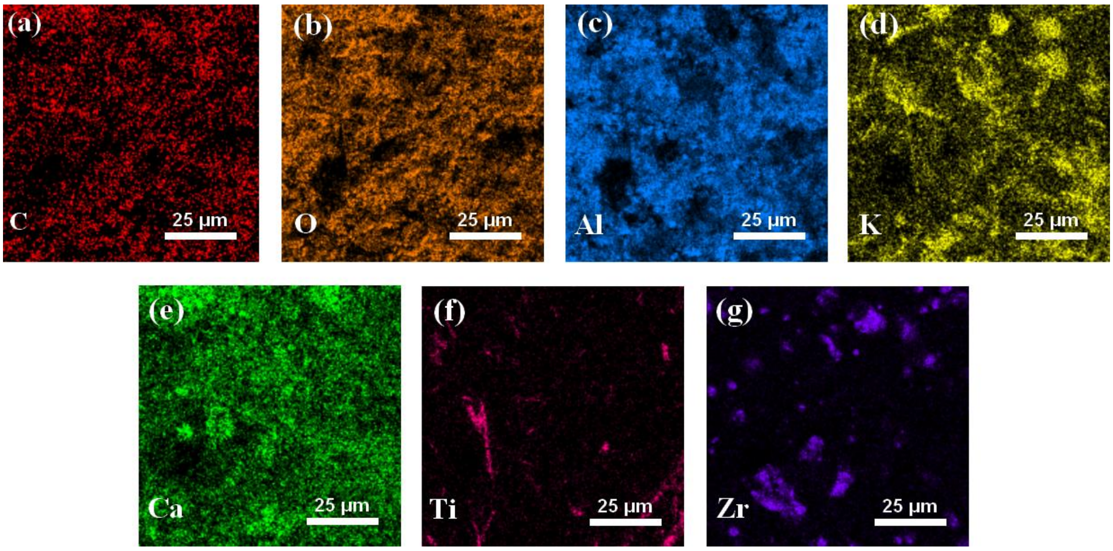

Figure S5. Element distribution of the modified separator before cycle testing of ZAB: (a) C, (b) O, (c) Al, (d) K, (e) Ca, (f) Ti and (g) Zr.

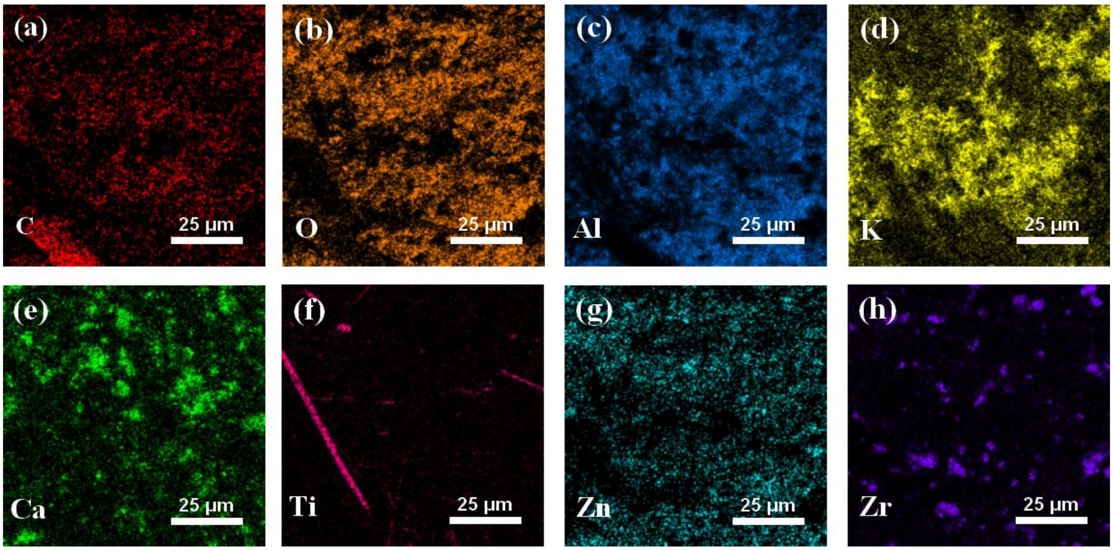

Figure S6. Element distribution of the modified separator at the side contacting with Zn anode after cycle testing of ZAB: (a) C, (b) O, (c) Al, (d) K, (e) Ca, (f) Ti, (g) Zn and (h) Zr.

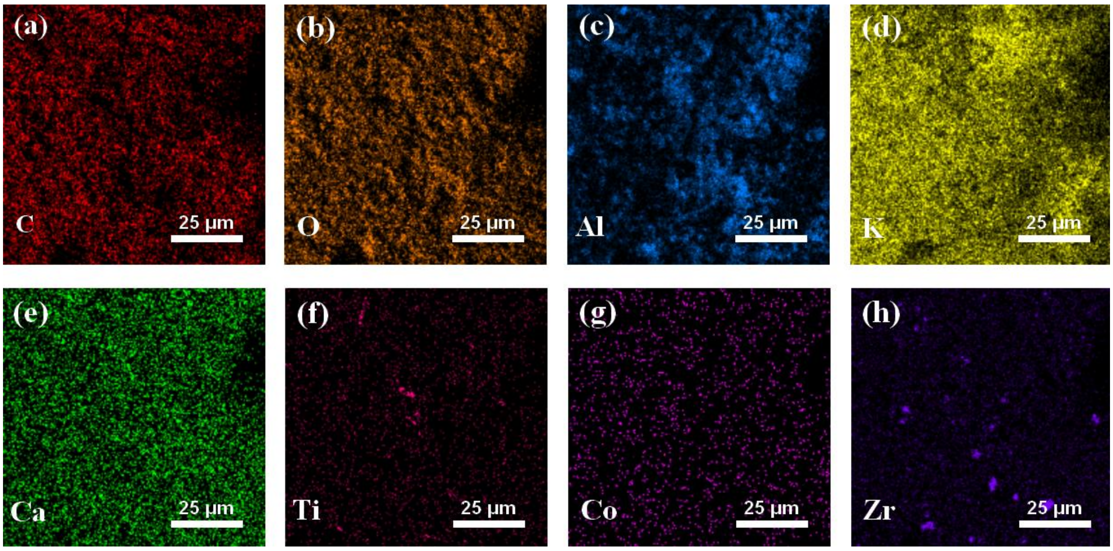

Figure S7. Element distribution of the modified separator at the side contacting with air cathode after cycle testing of ZAB: (a) C, (b) O, (c) Al, (d) K, (e) Ca, (f) Ti, (g) Co and (h) Zr.

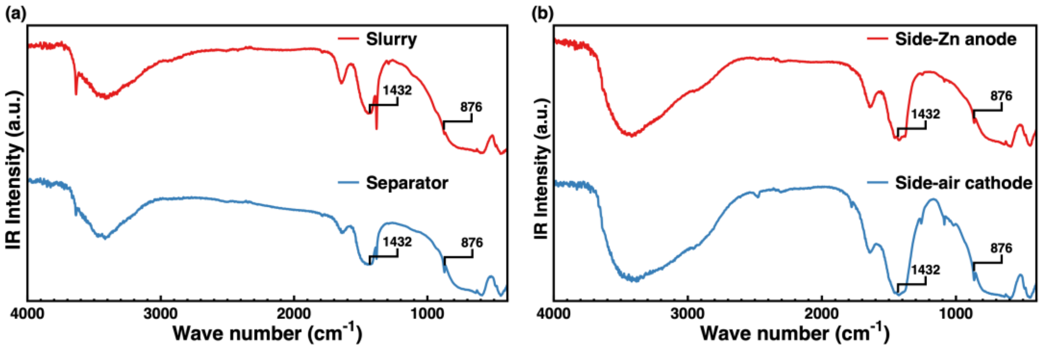

Figure S8. FT-IR patterns of the modified separator (a) before and (b) after cycle testing of ZAB.

Table S1. Element content of Zn anode after cycle testing of ZAB with modified electrolyte.

Element	wt%	At%
C K	14.06	30.25
O K	26.80	43.28
K K	11.59	7.66
Ca K	0.11	0.07
Zn L	47.43	18.74
Total	100.00	100.00

Table S2. Element content of Zn anode after cycle testing of ZAB with modified separator.

Element	wt%	At%
C K	13.48	28.59
O K	25.70	40.92

K K	25.45	16.58
Ca K	0.53	0.33
Zn L	34.84	13.58
Total	100.00	100.00

Table S3. Element content of air cathode after cycle testing of ZAB with modified electrolyte.

Element	wt%	At%
C K	14.15	24.25
O K	41.21	53.01
K K	35.83	18.86
Ca K	4.93	2.53
Zn L	3.88	1.35
Total	100.00	100.00

Table S4. Element content of air cathode after cycle testing of ZAB with modified separator.

Element	wt%	At%
C K	37.41	55.80
O K	26.02	29.14
K K	24.92	11.42
Ca K	0.66	0.30
Co K	10.99	3.34
Total	100.00	100.00

Table S5. Element content of the modified separator before cycle testing of ZAB.

Element	wt%	At%
C K	10.44	18.34
O K	36.47	48.10

Al K	29.47	23.05
K K	7.61	4.11
Ca K	8.05	4.24
Ti K	1.56	0.69
Zr L	6.40	1.48
Total	100.00	100.00

Table S6. Element content of the modified separator at the side contacting with Zn anode after cycle testing of ZAB.

Element	wt%	At%
C K	10.31	18.79
O K	33.53	45.84
Al K	25.48	20.66
K K	16.89	9.45
Ca K	4.50	2.46
Ti K	1.62	0.74
Zn L	2.38	0.80
Zr L	5.29	1.27
Total	100.00	100.00

Table S7. Element content of the modified separator at the side contacting with air cathode after cycle testing of ZAB.

Element	wt%	At%
C K	18.03	29.60
O K	36.21	44.62
Al K	12.23	8.93
K K	31.34	15.80
Ca K	1.99	0.98
Ti K	0.18	0.07
Co K	0.02	0.01
Zr L	0.00	0.00
Total	100.00	100.00
